# Making the Most of the Host; Targeting the Autophagy Pathway Facilitates *Staphylococcus aureus* Intracellular Survival in Neutrophils

**DOI:** 10.3389/fimmu.2021.667387

**Published:** 2021-06-16

**Authors:** Emilio G. Vozza, Michelle E. Mulcahy, Rachel M. McLoughlin

**Affiliations:** Host-Pathogen Interactions Group, School of Biochemistry and Immunology, Trinity Biomedical Sciences Institute, Trinity College Dublin, Dublin, Ireland

**Keywords:** *Staphylococcus aureus*, intracellular survival, autophagy, neutrophils, apoptosis

## Abstract

The success of *Staphylococcus aureus* as a human commensal and an opportunistic pathogen relies on its ability to adapt to several niches within the host. The innate immune response plays a key role in protecting the host against *S. aureus* infection; however, *S. aureus* adeptness at evading the innate immune system is indisputably evident. The “Trojan horse” theory has been postulated to describe a mechanism by which *S. aureus* takes advantage of phagocytes as a survival niche within the host to facilitate dissemination of *S. aureus* to secondary sites during systemic infection. Several studies have determined that *S. aureus* can parasitize both professional and non-professional phagocytes by manipulating the host autophagy pathway in order to create an intracellular survival niche. Neutrophils represent a critical cell type in *S. aureus* infection as demonstrated by the increased risk of infection among patients with congenital neutrophil disorders. However, *S. aureus* has been repeatedly shown to survive intracellularly within neutrophils with evidence now supporting a pathogenic role of host autophagy. By manipulating this pathway, *S. aureus* can also alter the apoptotic fate of the neutrophil and potentially skew other important signalling pathways for its own gain. Understanding these critical host-pathogen interactions could lead to the development of new host directed therapeutics for the treatment of *S. aureus* infection by removing its intracellular niche and restoring host bactericidal functions. This review discusses the current findings surrounding intracellular survival of *S. aureus* within neutrophils, the pathogenic role autophagy plays in this process and considers the therapeutic potential for targeting this immune evasion mechanism.

## Introduction

*Staphylococcus aureus* has evolved with the human immune system in order to survive as a commensal organism as well as a causative agent of disease. *S. aureus* is one of the most frequent causes of bloodstream infection worldwide, with a high hospital mortality rate ranging from 15-40% and a significant risk of reoccurrence in susceptible individuals (1, 2). Complications of *S. aureus* bacteraemia include secondary or metastatic infection, sepsis and septic shock (3). Metastatic infection by *S. aureus* is defined as infection at a secondary anatomical site that is distinct from the primary site of infection. Metastatic infections that occur during *S. aureus* bacteraemia include infective endocarditis, septic arthritis and vertebral osteomyelitis with reported prevalence ranges from 5.7-75.3% (4). Intracellular survival of *S. aureus* within host phagocytes has been highlighted as a mechanism employed by *S. aureus* to achieve metastatic infection and host dissemination (5–7).

In 2011, Thwaites et al. presented the idea that polymorphonuclear neutrophils (PMN) may represent a “privileged site” for *S. aureus* and may act as a Trojan horse for intracellular survival and dissemination to secondary sites in the host following an initial focus of infection (8). Evidence of this already existed in murine PMN (9); however, it has since been proven that *S. aureus* can survive intracellularly within PMN in murine and zebrafish *in vivo* models of infection as well as in primary human neutrophils (7, 10–12). Whilst much remains to be understood about how *S. aureus* achieves this, a key mechanism for intracellular survival within PMN has been identified. The autophagy pathway, a homeostatic cellular process of nutrient recycling within the cell, is co-opted by *S. aureus* as an intracellular survival niche (13). This review discusses what we now know about how *S. aureus* survives within PMN, since the initial opinion of Thwaites et al. that PMN act as a Trojan horse for *S. aureus*. We highlight the recent advances in uncovering the autophagy pathway as a conduit for intracellular survival. Furthermore, we speculate how this mechanism, that is subverted by *S. aureus*, could in-turn potentially be subverted by us as a novel approach to the treatment of *S. aureus* disease.

## *S. aureus* Is a Capable Facultative Intracellular Pathogen

Historically, *S. aureus* has been classed as an extracellular pathogen, with uptake and phagocytosis simply considered a consequence of the host immune response. However, the concept of *S. aureus* as a facultative intracellular pathogen, capable of invading, surviving and proliferating within non-professional and professional phagocytes has been consistently shown (6, 14–16), with intracellular survival now accepted as an active mechanism of immune evasion employed by this bacterium. Several *in vitro* studies have shown that intracellular survival of *S. aureus* can be achieved in a wide array of cell types (**Table 1**). However, the exact mechanism and life cycle of intracellular *S. aureus* is not fully understood and most likely varies in a strain and host cell dependant manner. Commonalities among many *in vitro* studies do highlight three potential key mechanisms; 1) The survival of *S. aureus* within acidified phagosomes potentially inducing bacterial stress responses that delay or perturb phagolysosome formation, 2) The formation of quasi dormant single colony variants (SCVs) and persisters resistant to killing and sensing by the host, 3) The induction of host autophagy and survival within host autophagosomes. However, it is likely that some overlap and redundancy exists among these mechanisms to enable intracellular survival.

**Table 1:** Intracellular survival of *S. aureus* has been shown across a range of cell types and involving multiple mechanisms.

Cell type	Strain	Mechanism	Ref.
Phagocytes			
BMDCs	PS80	Accumulation of autophagosomes permits intracellular survival and replication.	(10)
Murine alveolar macrophages MH-S	Newman	Delayed acidification of phagosomes and persistence inside acidic compartments.	(17)
THP-1	USA300 LAC	Survival within acidic phagosomes that increase *agr* expression and perturb phagolysosome formation.	(18)
RAW264.7	Mu3	Vancomycin resistance regulator VraR enhances the induction of autophagy for intracellular survival.	(19)
	USA300 LAC Newman	Growth observed in phagolysosomes dependent on acidic pH and the bacterial GraXRS regulatory system.	(20)
Human Monocyte derived Macrophages	Newman	Viable and dividing bacteria observed within vacuoles days after infection leading to host cell lysis.	(14)
Human PMN	USA300 LAC PS80	*S. aureus* resides in autophagosomes and delays apoptosis enabling prolonged intracellular survival.	(21)
Larval Zebrafish Neutrophils	SH1000	LC3-associated phagocytosis enables intracellular survival within non-acidified phagosomes.	(13)
Epithelial cells			
A459	Cowan	SCV and non-replicating persister formation in response to low pH of phagolysosomes enables persistence.	(22)
HaCaT	USA300 LAC	Survival observed in cytosol and autophagosomes. Selective advantage gained for *agr* mutants *via* autophagic suppression of the inflammasome.	(23)
HeLa	USA300 JE2	Intracellular survival, phagosomal escape and cytotoxicity dependent on multiple bacterial proteins.	(24)
293T	USA300 LAC 6850	Uses PSMα to escape host phagosome leading to bacterial replication in the host cytosol.	(25)
Endothelial cells			
EA.hy926	USA300 LAC	Replication within the host cytoplasm followed by cell lysis and escape. Non-dividing bacterial cells also observed intracellularly.	(26)
HUVECs	6850	SCVs fail to induce inflammatory response and persist intracellularly for several days.	(27)
Bone cells			
Murine Osteoclasts	USA300 LAC	Low intracellular bacterial number colocalized with phagolysosome whilst high bacterial number colocalized within non-acidic compartments.	(28)
Human osteocyte-like cells	WCH-SK2	Rapid generation of SCVs after intracellular invasion.	(29)

Impressively, several studies have reported the ability of *S. aureus* to survive intracellularly within dendritic cells, macrophages and neutrophils, phagocytic cells critical in the defence against and killing of invading pathogens (7, 10, 12, 13). *S. aureus* survival within macrophages has now been well-documented in studies using macrophage cell lines and primary cells. *S. aureus* was shown to survive over 24h in murine macrophage cell lines (17, 30) and up to 3-4 days in human monocyte-derived macrophages (14). Furthermore, live *S. aureus* has been observed to perturb phagolysosomal formation and reside within acidified phagosomes of THP-1 macrophages (18). When treated with phagosomal acidification inhibitors, viable intracellular *S. aureus* was dramatically reduced indicating *S. aureus* can manipulate host phagosomes for its own gain. In one particular study, alpha toxin (hla), produced by intracellular *S. aureus*, was shown to prevent mitochondrial recruitment to host phagosomes impairing ROS mediated killing within human monocytes and BMDMs (31), implicating perturbation of host bactericidal mechanisms in maturing phagosomes as the intracellular survival strategy of *S. aureus* whilst also implicating hla. *In vivo*, Kupffer cells localised to the liver were found to be the primary reservoir for *S. aureus* in a murine model of systemic infection (7). However, the complete removal of Kupffer cells using clodronate liposomes led to a reduction in *S. aureus* sequestration in the liver, persistent bacteraemia and 100% mortality indicating their importance in controlling infection. Regardless of their importance, intracellularly viable *S. aureus* was found to replicate within these Kupffer cells inside LAMP1-decorated acidic compartments with several compartments failing to generate superoxide. This model indicates that *S. aureus* survives within perturbed phagolysosomes of Kupffer cells as it’s intracellular niche before overwhelming and lysing the host cell. After cell lysis, extracellular *S. aureus* was then rapidly phagocytosed by infiltrating PMN. A follow up to this study showed that upon release from Kupffer cells, *S. aureus* disseminated to the peritoneal cavity and resided within GATA6^+^ macrophages for up to 2 days (32). This ultimately led to further dissemination to peritoneal organs which was reduced in mice lacking GATA6^+^ large peritoneal macrophages.

Two important virulence regulators have been heavily implicated in the intracellular survival of *S. aureus* within phagocytes; the *accessory gene regulator* (*agr*) and to a lesser extent, the *staphylococcal accessory element* (*Sae*) (10, 33–36). Both regulators govern the vast majority of cytolytic toxins produced by *S. aureus*, and when absent, lead to significant reduction in phagosomal escape as well as reduced intracellular burden. Additionally, the *agr* locus has also been shown to activate the *Sae* locus which may highlight a synergistic or overlapping relationship between the two regulators and their associated factors (37). An initial study of intracellular survival within human monocyte derived macrophages (hMDMs) highlighted the importance of the *agr* locus and the stress response and virulence regulator *SigB*, as the absence of either regulator led to significant reduction in intracellular burden and cell death (14). More recently, the *agr* and *Sae* locus have been further implicated in intracellular survival as mutant strains of either regulator showed significant reductions in phagosomal escape within THP-1 cells and hMDMs, with double mutants showing the greatest reduction in cytotoxicity and phagosomal retention (35). These results were attributed to a group of cytolytic peptides regulated by the *agr* locus and the *Sae* locus; Phenol Soluble Modulin (PSM) α, Leucocidin A/B (lukAB) and Panton–Valentine leucocidin (PVL). The importance of the *agr* locus in intracellular survival has been demonstrated using the Nebraska transposon mutant library and the CA-MRSA strain JE2. Single mutants for *agr*A, *agr*B, *agr*C and PSM transporter *pmt*C showed the greatest reduction in phagosomal escape within Hela cells which potentially mimics the mechanism found in phagocytes (24). Grosz et al., showed that phagosomal escape of the MRSA strains LAC USA300 and MW2 USA400 as well as the MSSA strain 6850 was exclusively dependent on PSMα (25). This was determined using mutant strains of PSMα which failed to escape host phagosomes in professional and non-professional phagocytes with PSMα complementation restoring phagosomal escape. Surewaard et al., showed that PSMs are functionally inhibited by serum lipoproteins with human serum, which protect human PMN from PSM induced cell lysis (38). This implies that in the body, serum proteins render PSMs redundant in the extracellular environment which the authors theorize implicate the role of PSMs to be found intracellularly. Indeed, the authors showed that PSMα expression was induced between 45 minutes to 2 hours post phagocytosis within human PMN implicating PSMs in the intracellular environment.

Another important mechanism employed by *S. aureus* to establish intracellular survival is the formation of SCVs which are a slow growing bacterial phenotype that exhibit an altered and reduced metabolism compared to WT colonies (39). SCVs also show enhanced antimicrobial resistance and have long been associated with chronic and recurrent infection (40). The formation of SCVs represents an important immune evasion mechanism in sustaining long term intracellular survival that is particularly evident in non-professional phagocytic cell types. However, SCVs can form within phagocytic cell types but their formation has been shown to reduce their intracellular survival within phagocytes compared to WT colonies. For instance, THP-1 macrophages have been shown to clear SCVs more efficiently than wild type counterparts suggesting SCV formation is disadvantageous in phagocytes (41). SCVs can persist up to a week post infection within human osteoblasts and the endothelial HUVEC cell line in contrast to human macrophages which become undetectable 3 days post infection (42). SCVs show a marked reduction in *agr* expression, due to their altered metabolic phenotype, and show a greater reliance on *SigB* as mutants of this virulence factor show a significant decrease in persistence (39, 43, 44). Tuchscherr et al., showed that *SigB* is crucial in the formation of SCVs and intracellular persistence within human osteoblasts (45). Loss of *agr* and *SarA* expression led to increased persistence within osteoblasts whilst significantly reducing cytotoxicity against human and murine PMNs. However, each of these virulence regulators were shown to be important *in vivo* using both an acute and chronic murine model of infection (4 days vs 14 weeks post infection). Whilst *SigB* was crucial for establishment of SCVs and their associated long-term persistence, phagosomal escape was dependent on the *agr* and *SarA* loci. Whilst the *agr* locus is critical in subverting phagocytes, likely through subverting bactericidal mechanisms such as phagolysosomal formation, it’s absence in SCVs enables long term survival in non-professional phagocytes which potentially renders SCVs susceptible to killing by phagocytes.

## Autophagy Is a Key Mechanism for the Intracellular Survival of *S. aureus*

**Figure 1:**
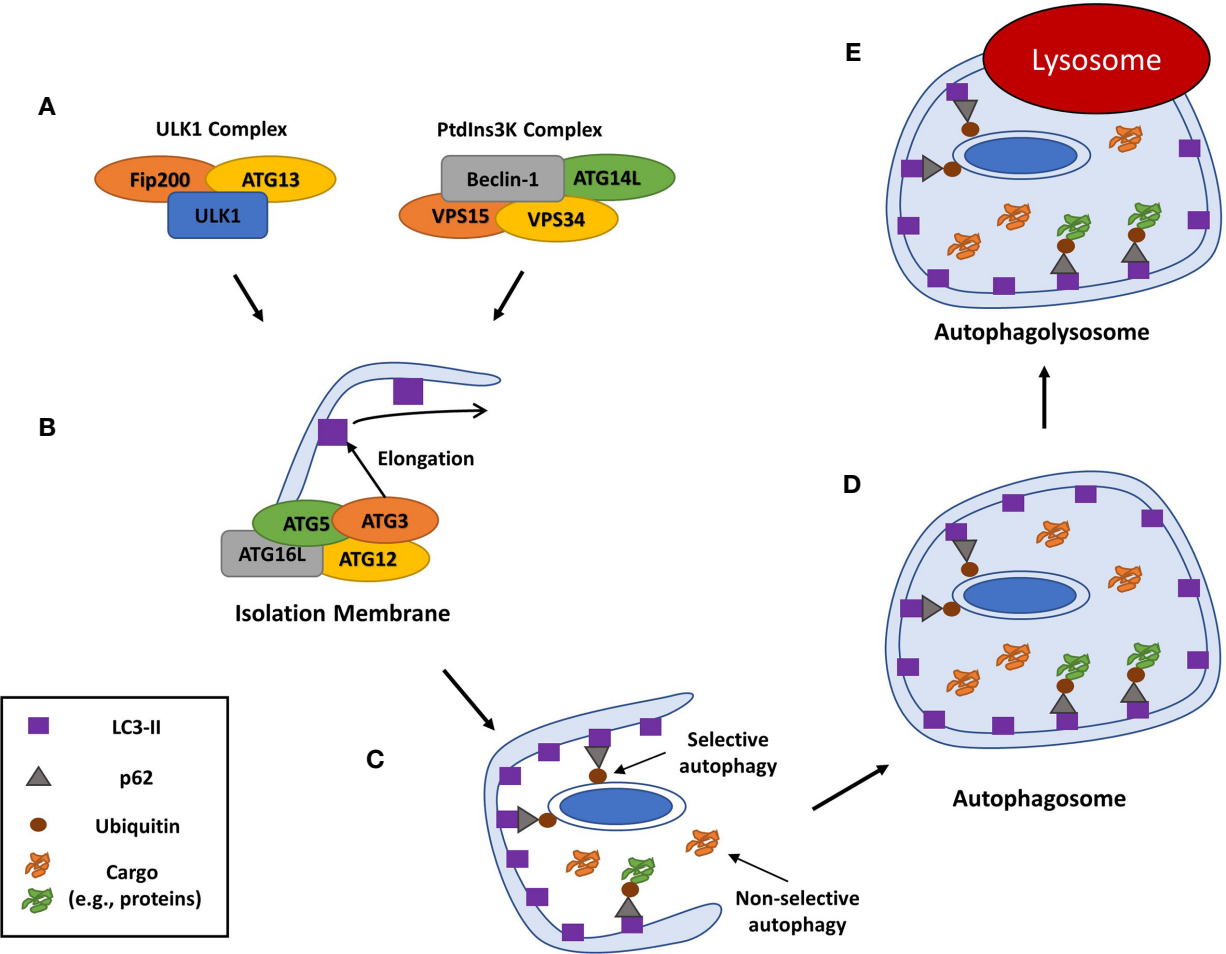
Overview of mammalian autophagy. Autophagy is induced through the recruitment of numerous autophagy related proteins (ATGs) to a specific cytoplasmic location. This is followed by the formation of the isolation membrane that eventually elongates and closes around organelles or cell debris to be digested to form a double-membraned phagosome, or autophagosome (46). **(A)** The autophagic cascade is initiated by core molecular machinery including the Ulk1 complex and an activation complex comprising of Beclin-1, the class III PI3-kinase, Vacuolar protein sorting 34 (VPS34), ATG14L and VPS15 (47). **(B)** This complex facilitates the *de novo* formation of an isolation membrane that then elongates to form a double membraned phagophore, by recruiting several ATGs, including ATG3, ATG16L and the ATG-12-ATG5 complex, which then convert the ATG8 protein LC3-I to its lipidated, membrane-bound form LC3-II (48). **(C)** As well as non-selective autophagy of cytoplasmic components, cargo can be specifically recognized and marked for autophagy typically by ubiquitination. Ubiquitinated cargo is then recognized by cargo receptor molecules including SQSTM1/p62, CALCOCO2 and OPTN and trafficked to the phagophore through an interaction between the cargo receptor and LC3 (49–51). **(D)** Continued LC3-II conjugation to the phagophore causes it to elongate and form a cargo containing autophagosome which is trafficked to the host lysosome. **(E)** The autophagosome matures and fuses with a lysosome, the autophagic cargo is degraded and the nutrients are released back into the cytosol to be re-used by the cell.

Intracellular survival of *S. aureus* is a remarkable feat in which *S. aureus* can thrive in phagocytic and non-phagocytic cells by perturbing phagolysosomes and inducing SCV formation, respectively. However, a third mechanism has emerged in recent years involving host macroautophagy (herein called autophagy) that can occur within professional and non-professional phagocytes in an *agr* dependent or independent fashion depending on the cell type. This mechanism has been shown to affect both SCVs and WT colonies implicating the manipulation of autophagy as a critical and key step in establishing intracellular survival across various cell types and strains. Autophagy is a conserved eukaryotic homeostatic process in which damaged cellular organelles are recycled during cellular stress or starvation in order to create a supply of nutrients for cell survival (**Figure 1**) (52). As well as its role in homeostasis, autophagy also plays a part in the innate immune response to infection by selectively engulfing invading bacteria in a specialized autophagic process of pathogen clearance called Xenophagy (53). The xenophagic response involves the formation of an autophagosome to engulf intracellular bacteria and traffic them towards lysosomal degradation and is an important process in controlling intracellular replication of pathogens including *Listeria monocytogenes*, *Salmonella enterica* serovar Typhimurium and *Mycobacterium tuberculosis* (54–56). However, these pathogens as well as many other pathogens such as *Escherichia coli* have been reported to manipulate or subvert the autophagic pathway for survival (57–61). Moreover, many studies have described divergent mechanisms for *S. aureus* intracellular survival and replication using the autophagic pathway in both non-professional and professional phagocytes, with several autophagy inhibitors such as VPS34-IN1 and 3-methyladenine shown to reduce intracellular survival of *S. aureus* across a range of cell types (10, 21, 62, 63).

In a study by Schnaith et al., *S. aureus* survived and replicated in autophagosomes within HeLa cells (33). *S. aureus* was rapidly taken up by Rab7-positive endophagosomes and became enveloped in double-membraned autophagosomes within 3h of infection. *S. aureus*-harbouring phagosomes were mostly LC3-decorated and underwent reduced acidification. Activation of autophagy with rapamycin treatment led to a higher bacterial load within cells whereas conversely, autophagy inhibition using wortmannin reduced the bacterial burden, indicating that *S. aureus* replication depends on an activated autophagic pathway. Intracellular survival was followed by eventual escape into the cytoplasm coupled with caspase-independent, ATG5-dependent host cell death with high levels of vacuolization, indicative of autophagic cell death. Recently, the induction of autophagy in HeLa cells by *S. aureus* was compared to *Salmonella* infection, which is known to evade the autophagy pathway to facilitate intracellular survival (62, 64). While both bacteria showed an ability to survive intracellularly, *S. aureus* escaped from lysosomal compartments with less lysosomal membrane damage compared to *Salmonella*, suggesting that *S. aureus* may actively and specifically subvert the xenophagy defence pathway. Blocking autophagy mediated by the Ulk1 complex made cells more resistant to *S. aureus* infection compared to *Salmonella* infection which showed no change. Furthermore, Ulk1 inhibition reduced the ability of *S. aureus* to survive intracellularly, suggesting that an autophagy-dependent niche is essential for intracellular survival. This study also highlighted the importance of the *agr* locus as accumulation of LC3-II and p62 aggregates was shown to be partially dependent on a functional *agr* locus. In another study, changes to the host central carbon metabolism during *S. aureus* intracellular survival was evident in HeLa cells infected with MRSA strain USA300 (65). *S. aureus* infection led to depletion of glucose and amino acid pools as well as increased levels of glutaminolysis and activation of starvation-induced autophagy. Interestingly, although autophagy was activated by *S. aureus* infection, no bacteria were found within autophagosomes. This study highlights an alternative or additive mechanism of autophagy-mediated intracellular survival whereby *S. aureus* is manipulating the autophagy pathway in order to aid it in scavenging nutrients within the cellular space. In a study by Neumann et al., the autophagic response was induced by *S. aureus* infection of murine fibroblasts (66). *S. aureus* was encapsulated in LC3-positive phagosomes and multilamellar membranes were identified using TEM indicative of autophagosomes. In this model, intracellular *S. aureus* was ubiquitinated shortly after invasion and then co-localized with the trafficking proteins p62, ubiquitin and LC3 as well as trafficking proteins OPTN and CALCOCO2 indicating direct targeting of *S. aureus* by host autophagy. No acidification of phagosomes was reported, and *S. aureus* was eventually able to escape to the cytosol where bacterial replication was evident. In CHO cells, exposure to *S. aureus* also led to intracellular survival within non-acidic, LC3-labelled compartments and eventual escape into the cytosol (67). In a separate study using CHO cells, *S. aureus* induced the formation of LC3-decorated tubules from *S. aureus*-harbouring phagosomes (68). These tubules were observed to facilitate efficient bacterial replication.

In professional phagocytes, *S. aureus* can evade killing by murine bone marrow-derived dendritic cells and macrophages by manipulating the autophagy pathway in a strain-specific mechanism that appears to be dependent upon expression of the *agr* locus (10). Increased cytolytic protein production was associated with a disruption in autophagic flux, leading to autophagosomal accumulation and eventual cell lysis. The ability to survive by manipulating the autophagic pathway correlated with persistence in an *in vivo* model of systemic infection. Induction of host autophagy by *S. aureus* has also been reported in RAW264.7 macrophages. *S. aureus* expressing the vancomycin resistance-associated sensor/regulator, VraSR, were shown to enhance gene expression of beclin-1 and ATG5 compared to an isogenic VraSR mutant. The VraSR mutant also showed increased turnover of LC3-II and p62 indicating reduced disruption of autophagic flux as well as significantly reduced intracellular burden compared to the WT and VraSR complemented mutant strains (19). In bovine macrophages, *S. aureus* was capable of inducing host autophagy which was marked by significant increases in LC3-II and increased visualisation of host autophagosomes versus uninfected cells using TEM imaging and confocal microscopy (63). Importantly, p62 levels were elevated yet p62 degradation, a consequence of autophagosomal degradation and autolysosomal formation, was arrested indicating a disruption in autophagic flux. Furthermore, treatment with the autophagy inhibitor 3MA led to significant decreases in intracellular *S. aureus*. Overall, the manipulation of the autophagy pathway plays a key role in the intracellular survival of *S. aureus* across multiple cell types enabling enhanced survival and replication within the host cell.

## Intracellular Survival of *S. aureus* Within Neutrophils

**Figure 2:**
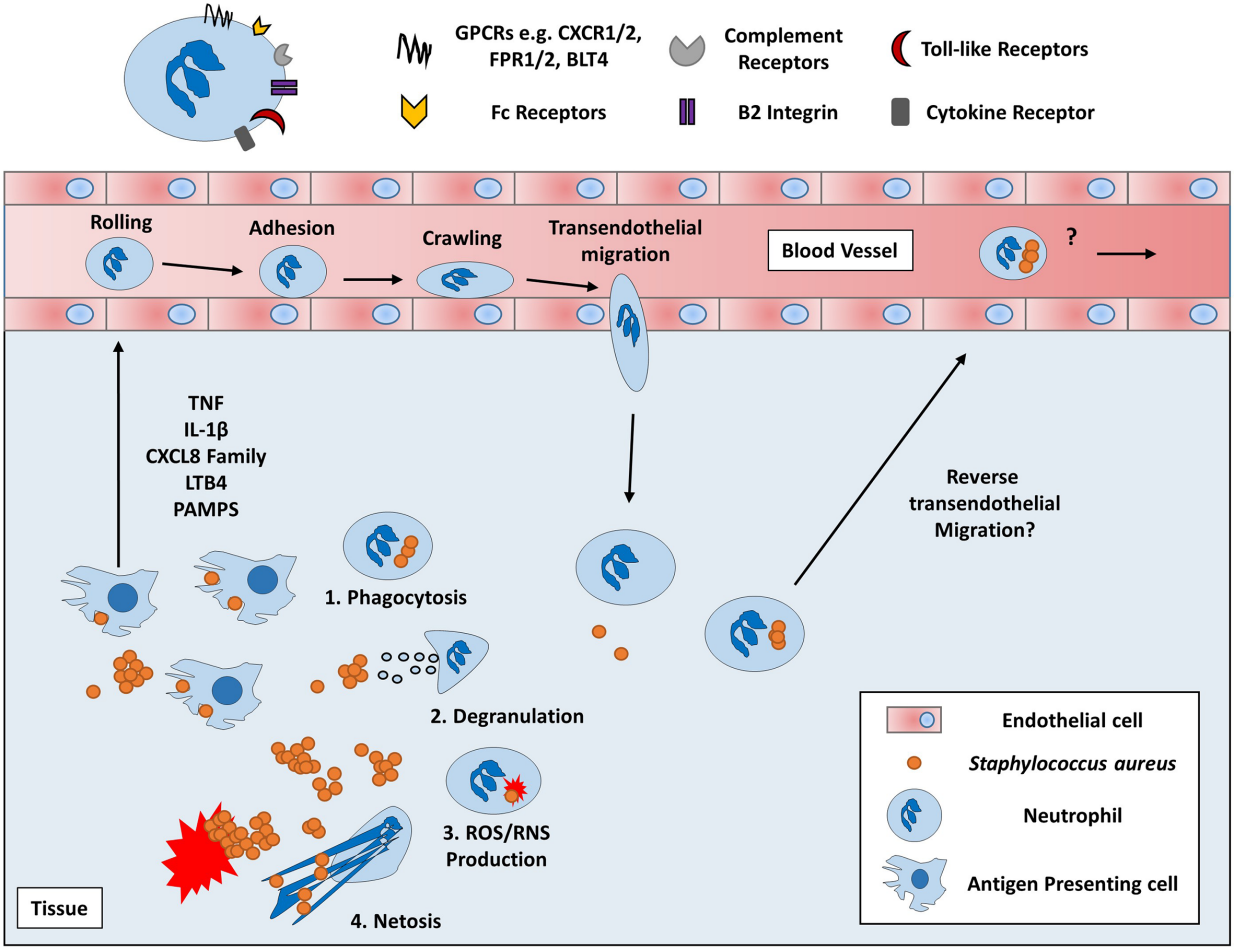
Overview of Neutrophil chemotaxis and effector functions. Neutrophils are circulatory immune cells which respond to host or pathogen derived chemoattractants using various receptors such as chemokine receptors (e.g. CXCR2) and pathogen recognition receptors (e.g. FPR2) (69, 70). Proinflammatory cytokines, chemokines, and lipid mediators, such as TNF, CXCL8 and leukotriene B_4_ (LTB_4_) respectively, are produced by local immune and non-immune cells which recruit PMN from the peripheral blood (71, 72). Initially, PMN slow their passage through the blood *via* weak interactions with the endothelium causing PMN to roll along the surface of the blood vessel. PMN then begin to adhere to the endothelium through stronger B_2_ integrin interactions *via* I-CAM1 molecules expressed on the surface of endothelial cells (73). Ultimately, PMN crawl across the endothelium toward the chemotactic gradient until they traverse through the blood vessel to the site of infection *via* transendothelial migration. Once at the site of infection, PMN begin to exert their various effector functions which include 1) phagocytosis *via* complement and Fc receptors to eliminate *S. aureus via* several intracellular mechanisms 2) releasing primary, secondary and tertiary granules containing antimicrobial peptides such as cathepsins, defensins and gelatinases into the extracellular space or phagosomal compartment, 3) Generating reactive oxygen or nitrogen species *via* NADPH oxidase, myeloperoxidase and nitric oxide synthase and 4) the release of uncondensed chromatin imbued with histones and antimicrobial peptides in a process of cell death known as NETosis (74, 75). Despite these effective killing mechanisms, *S. aureus* can survive intracellularly within PMN and could potentially induce rTEM in PMN to enter the peripheral blood and disseminate to secondary sites (6, 8, 73).

Phagocytes are critical in the defence against *S. aureus*, yet amazingly *S. aureus* has developed the ability to co-opt these cells for its own survival with PMN representing the most unlikely target for intracellular survival. PMN are pivotal in the innate immune response against *S. aureus* infection. Neutrophils are recruited from the systemic circulation to the site of infection in response to several chemotactic factors such as chemokines and pathogen associated molecular patterns (PAMPS) where they then recognise invading bacteria *via* opsonised and non-opsonised mechanisms (**Figure 2**). *S. aureus* is engulfed and ultimately killed by several methods utilized by PMN including degranulation, the production of reactive oxygen species and the secretion of antimicrobial peptides (74). Additionally, extracellular *S. aureus* can also be killed through the production of NETs (75). The importance of PMN in *S. aureus* infection is most evident in individuals with deficiencies in PMN activity. Individuals with chronic granulomatous disease, a deficiency in NADPH oxidase, suffer from recurrent *S. aureus* infections (76, 77). In addition, frequent *S. aureus* infections are a consequence of severe congenital neutropenia, Chediak-Higashi syndrome and leukocyte adhesion deficiency 1 (78, 79). Furthermore, evidence from murine *in vivo* models have shown that neutrophil depletion leads to increased bacterial burden within the liver after intravenous injection as well as increased mortality after intratracheal injection (11, 80). Such observations highlight the importance of PMN in controlling *S. aureus* infection and the consequence of their absence which results in overwhelming disease.

Although optimal PMN function is vital to protect the host against overwhelming bacterial burden during *S. aureus* infection, high levels of PMN recruitment can be detrimental. Limiting PMN migration during a murine model of lethal *S. aureus* infection led to a decrease in mortality and a reduced bacterial burden at the infection site, whereas repletion with PMN reversed this protective effect (9). Heightened CXC-chemokine driven PMN recruitment has been shown to exacerbate pathogenesis in a murine model of wound infection (81). Furthermore, IFNγ-/- mice displayed a decrease in bacterial burden at the site of infection due to reduced CXC chemokine production and subsequent PMN recruitment (82). An IFNγ-dependent increase in PMN recruitment to the site of infection provided a PMN-rich environment in which *S. aureus *could survive more readily. More recently, during intraperitoneal infection in mice, *S. aureus* survived at significantly higher levels within PMN than in macrophages or dendritic cells and were found predominantly within PMN disseminated from the peritoneal cavity to the bloodstream (10). These studies allude to intracellular survival in PMN as a possible active bacterial virulence mechanism during infection which has now been demonstrated both *in vitro* and *in vivo*. *S. aureus* intracellular survival was observed in PMN after gentamicin treatment during murine intraperitoneal infection (9). Surviving bacteria were viable and PMN harbouring *S. aureus* could cause infection when administered to naïve mice. Moreover,* S. aureus *survival within murine PMN after gentamicin treatment was dependent on the expression of the SarA global regulator, suggesting that intracellular survival is an active immune evasion strategy. Excessive levels of both CXCL8 and LTB_4_ has been shown to repel PMN migration whilst recent studies have also observed reverse transendothelial migration (rTEM) of PMN from damaged tissues (73, 83). rTEM PMN have been identified as ICAM1^hi^CXCR1^lo^ expressing cells which have been detected in the peripheral blood of patients with chronic systemic inflammation (84). As such, this subpopulation of rTEM PMN could represent a key target of *S. aureus* to escape the primary site of infection and create secondary sites of infection.

Though the intracellular environment of neutrophils represents a harsh and dangerous space for bacteria to persist, *S. aureus* has amassed a large arsenal of factors to permit its intracellular survival. Neutrophils generate large amounts of reactive oxygen species (ROS) and reactive nitrogen species to effectively kill both extracellular and intracellular pathogens. To combat this, *S. aureus* has developed several protective enzymes to overcome free radicals, namely staphyloxanthin, catalase and superoxide dismutase (85, 86). These enzymes can effectively shield the pathogen enabling persistence during infection. Recently, a bacterial factor known as Staphylococcal peroxidase inhibitor (SPIN) was characterized and shown to inhibit myeloperoxidase (MPO), a key enzyme in neutrophils used to generate the bactericidal molecule hypochlorous acid (HOCL) (87). SPIN was shown to be heavily upregulated after phagocytosis by human PMNs leading to increased survival and resistance to neutrophil-derived ROS. This factor was shown to be regulated by the two-component regulatory system, *Sae*, a staphylococcal regulator previously implicated in intracellular survival of *S. aureus* (35).

Intracellular *S. aureus* recovered from PMN have been shown to upregulate RNAIII expression within the first hour post phagocytosis whilst extracellular *S. aureus* shows substantially less RNAIII expression (34). This is likely due to the confined space of intracellular compartments as confinement induced quorum sensing has been shown to increase *agr* activity due to the rapid accumulation of the autoinducing peptide (AIP) responsible for *agr* activation (88). Strains with a greater capacity to survive intracellularly within phagocytes show increased RNAIII expression whilst also showing greater cytolytic toxin production as measured by a vesicle lysis test (10). Whilst the *agr* locus is known to enhance PMN lysis, it was also been shown to enhance intracellular survival within PMN potentially in a similar fashion as observed in other phagocytes (21, 34).

## Manipulation of Autophagy Facilitates Intracellular Survival of *S. aureus* Within PMN

As the manipulation of autophagy by intracellular *S. aureus* has been shown in both macrophages and dendritic cells, it can easily be theorized that autophagy too plays a central role in the manipulation of PMN for intracellular survival. Recently it has been shown using a larval zebrafish model of *S. aureus* infection, that *S. aureus* survives within spacious LC3-positive phagosomes within both PMN and macrophages (13). However, the LC3 signal decreased over time for macrophages and not for PMN, suggesting a disruption in autophagic flux in PMN. Survival was dependent on a functioning NADPH oxidase, as Cyba/p22phox knock-down in larvae, as well as chemical depletion of ROS production using DPI, led to a near complete abolition of LC3-*S. aureus* association. This dependence on ROS production for LC3-*S. aureus* co-localization meant that survival was attributed to a mechanism called LC3-associated phagocytosis (LAP). LAP uses a portion of the autophagy machinery to conjugate LC3 to single-membraned phagosomes rather than double-membraned autophagosomes indicative of the canonical autophagy pathway (89). Loss of this pathway through an ATG5/ATG16L1 double knockdown led to an almost complete loss of LC3-*S. aureus* vacuoles and larvae were significantly more resistant to infection, suggesting that blocking LAP results in a loss of the *S. aureus* survival niche. Although p62, which is a marker for selective autophagy, also colocalized with *S. aureus* in this model, co-localization was downstream of LAP activation and was actually attributed to a host-protective mechanism in response to *S. aureus* infection, as loss of p62 led to increased host susceptibility to infection (66). Furthermore, p62 deficiency had no effect on the formation of LC3-positive phagosomes. This implies that *S. aureus* does not subvert canonical autophagy in zebrafish PMN but rather, takes advantage of single membraned LAPosomes to avoid killing. In contrast, *S. aureus* has been shown to survive within primary human PMN by subverting the autophagy pathway (21). *S. aureus* intracellular survival coincided with a disruption in autophagic flux as determined by the accumulation of the autophagic markers p62 and LC3-II which under normal autophagic flux undergo degradation upon fusion of the autophagosome and host lysosome. Replicating *S. aureus* was also observed within autophagosomes using TEM microscopy of infected human PMN. Furthermore, blockade of autophagosomal formation, with the autophagy inhibitors bafilomycin A1 and VPS34-IN1, led to a significant reduction in intracellular burden thus stressing the importance of the autophagy pathway in the intracellular survival of *S. aureus* within PMN.

**Figure 3:**
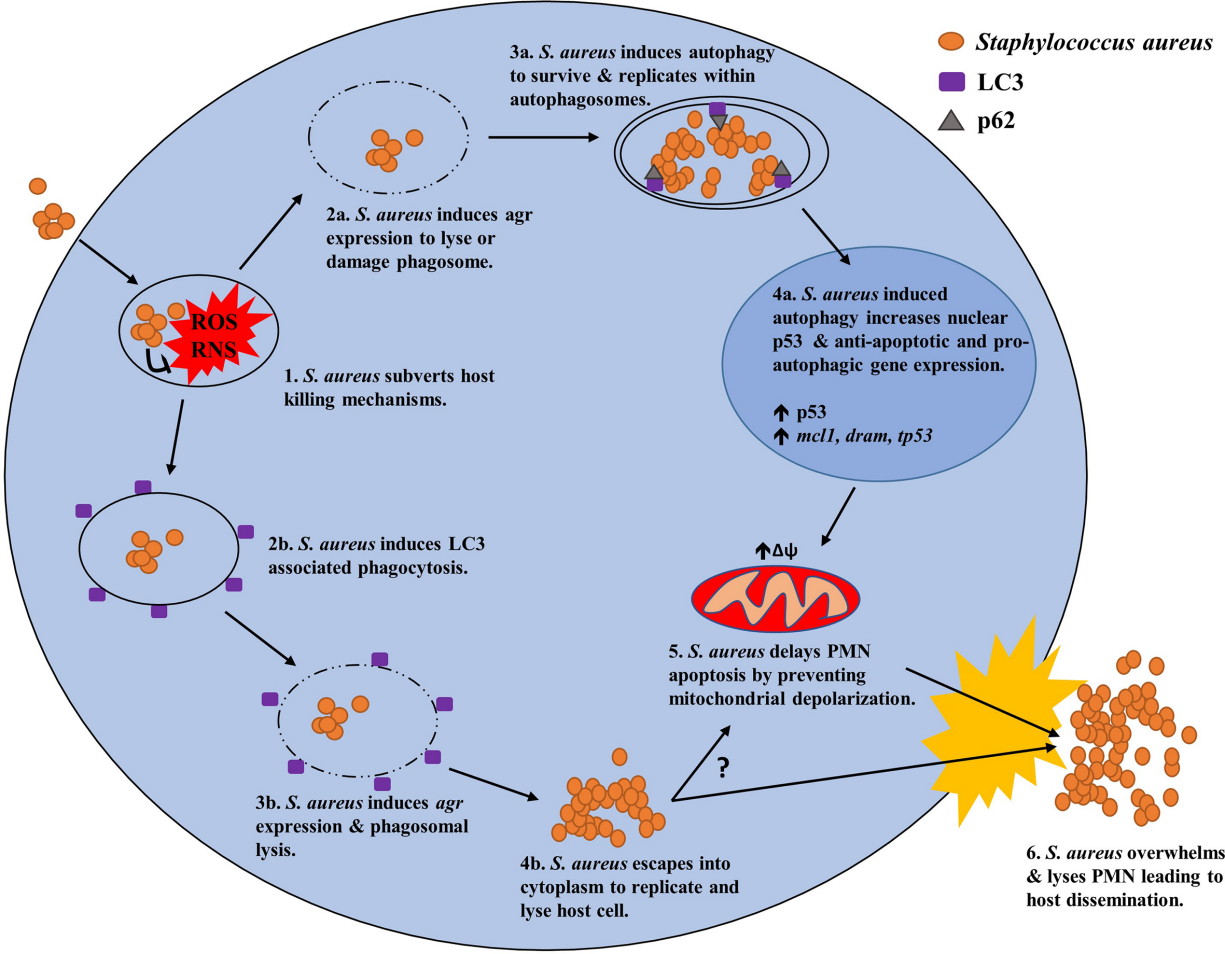
Potential mechanisms of *S. aureus* to manipulate host autophagy in PMN for intracellular survival. (1.) Upon phagocytosis by PMN, *S. aureus* subverts host killing mechanisms such as ROS generation and persists intracellularly by manipulating canonical or non-canonical (LAP) autophagy. (2a.) Using canonical autophagy, *S. aureus* lyses or damages the host phagosome using an *agr* regulated toxin. (3a.) Phagosomal lysis/damage induces host autophagy enabling its uptake into an autophagosome as its primary intracellular niche. (4a.) Induction of the canonical autophagy pathway leads to increased levels of nuclear p53 and anti-apoptotic and pro-autophagic gene expression of *mcl1* and *dram* respectively. (5.) Increases in anti-apoptotic factors prevents mitochondrial depolarization and subsequent induction of the apoptotic pathway resulting in prolonged PMN survival. (6.) Eventually, *S. aureus* exhausts its intracellular niche and overwhelms the host cell resulting in cell lysis and host dissemination. (2b.) Alternatively, *S. aureus* can utilize non-canonical autophagy *via* LAP were the bacterium resides within LC3 decorated phagosomes as its primary niche. (3b.) *S. aureus* uses an *agr* regulated toxin to avoid bacterial killing before lysing the LC3 decorated phagosome. (4b.) *S. aureus* then escapes into the cytosol where it then replicates. (5.) It is unknown whether this mechanism can lead to increased PMN survival however a link between the two may exist. (6.) Ultimately *S. aureus* overwhelms the host cell leading to cell lysis and host dissemination.

Altogether, two mechanisms utilising the autophagic machinery of PMN have thus far been identified to facilitate the intracellular survival of *S. aureus* (**Figure 3**). One mechanism relies on LAP to persist within LC3 decorated phagosomes as its primary niche, enabling the bacterium to avoid killing whilst actively replicating leading to eventual host cell lysis. The other mechanism relies upon canonical autophagy whereby intracellular *S. aureus* reside within autophagosomes as their primary niche in which they grow and divide presumably before overwhelming the host cell for dissemination. Remarkably, *S. aureus* is not only capable of subverting both canonical and non-canonical autophagy pathways for its own gain but may also act to disrupt the apoptotic pathway, presumably with the goal of protecting its intracellular niche.

## Increased PMN Survival by *S. aureus* Potentiates Intracellular Survival

Overall PMN represent an unlikely intracellular reservoir as mature PMN are terminally differentiated with a short circulating half-life (~7-19 hours in peripheral blood) controlled by constitutive apoptosis (90–92). However, several intracellular pathogens have been shown to delay apoptosis through the modulation of pro- and anti-apoptotic gene expression or cytokine production increasing PMN life span and thus protecting their intracellular niche (93–96). Ample evidence now exists that *S. aureus* does indeed survive within PMN enabling it to disseminate to secondary sites of infection. As such, any delay, even briefly, in PMN apoptosis would give *S. aureus *a survival advantage for long enough to potentially proliferate and disseminate, supporting the “Trojan horse” theory of *S. aureus* immune evasion.

Constitutive PMN apoptosis is primarily controlled by the Bcl-2 family of pro- and anti-apoptotic proteins. In PMN, the most prominent Bcl-2 family members include anti-apoptotic proteins Mcl-1 and A1, and pro-apoptotic factors such as Bid, Bim, Bad and Bax (97). Once apoptosis is initiated, PMN undergo several changes including rounding up of the cell, DNA fragmentation and phosphatidylserine exposure on the surface of the cell to facilitate macrophage efferocytosis. Under homeostatic conditions, circulating aged apoptotic PMN migrate to the liver, spleen and bone marrow were they are removed by Kupffer cells and other tissue resident macrophages (98, 99). Studies examining changes in primary human PMN during *S. aureus* infection *in vitro* reported that PMN rapidly display regular markers of apoptosis such as increased surface exposure of phosphatidylserine and loss of mitochondrial membrane potential (100). However, they also exhibited signs of a dysregulated apoptosis phenotype including sustained levels of PCNA, a lack of caspase-3 activation, increased surface expression of CD47 and inhibited macrophage efferocytosis. In other studies, the staphylococcal cell wall-associated factor lipoteichoic acid caused a delay in PMN apoptosis *in vitro* via TLR-2-mediated activation of NF-kB (101). The apoptotic fate of PMN during *S. aureus* infection was shown to be dependent on multiplicity of infection (MOI); low bacteria-PMN ratios could inhibit apoptosis whereas high ratios could induce it (102). The inhibition of PMN apoptosis in response to low MOI was shown to be cytokine dependent as blocking antibodies for IL-6 and TNF restored the apoptotic phenotype to that of uninfected PMN. The researchers speculated that high MOI of *S. aureus* leads to excessive TNF production that overrides the possibly anti-inflammatory effect of IL-6 seen in lowly infected PMN. These conflicting accounts of the changes to PMN lifespan during *S. aureus* infection, suggest that the apoptotic fate of *S. aureus* harbouring PMN may be context dependent.

The ability of *S. aureus* to delay apoptosis is a remarkable feat which suggests a pathogenic advantage is gained by the increased lifespan of infected PMN, presumably through the facilitation of intracellular survival. *S. aureus* has been shown to delay PMN apoptosis exemplified by a decrease in mitochondrial membrane permeabilization, a decrease in caspase-3 cleavage and lower levels of DNA degradation in *S. aureus*-harbouring PMN (21). Additionally, increases in the gene and protein expression of the anti-apoptotic factors Mcl-1 and A1/Bfl-1 was observed but no changes were found in the pro-apoptotic Bcl-2 member Bax. Crucially, this delay in the activation of the apoptosis pathway was dependent on an active autophagy pathway, as inhibition of autophagy activation with VPS34-IN1 led to an increase in DNA degradation and a reduction in expression of Mcl-1 (21). This close association between autophagy and apoptosis may be because these pathways share common effector molecules. For example, Mcl-1 is an anti-apoptotic homologue of the Bcl-2 family, but also plays a role in regulating autophagy by interacting directly with the critical autophagy protein beclin-1 (103). In neuronal cells, deletion of Mcl-1 leads to the induction of host autophagy implying Mcl-1 supresses autophagy likely through its binding of Beclin-1 (104). However, human neutrophils treated with tumour culture supernatants show increased survival and autophagy rates which is marked by the retainment of Mcl-1 implying a more complex relationship may exist (105). Mcl-1 is critical for PMN survival as PMN lack many Bcl-2 members and thus rely heavily on Mcl-1 for its anti-apoptotic function (106). However, Mcl-1 has a uniquely extremely high turnover rate in PMN compared to other members of the Bcl-2 family and so plays a key role in controlling PMN apoptotic cell death (106). In the context of intracellular *S. aureus* infection, much remains unclear as to the relationship between Mcl-1 and autophagy. However, during intracellular survival of *S. aureus* within PMN, inhibition of autophagy with VPS34-IN1 leads to significant reductions of Mcl-1 at the gene and protein level suggesting the induction of host autophagy leads to Mcl-1 mediated survival during *S. aureus* infection (21). Reduced gene expression of Mcl-1 under inhibition of autophagy was also accompanied by a significant reduction of the antiapoptotic genes *bcl2* and *bcl2a1*.

While *S. aureus* can expertly delay host apoptosis in PMN, several studies have also shown that it can induce apoptosis among other forms of cell death through the production of various virulence factors. For instance, PVL has been shown to induce apoptosis in PMN through a bax-independent process involving cytochrome c release and mitochondrial associated caspase-8 and -9 activation (107). Additionally, PVL production in PMN can lead to the induction of necroptosis, a regulated form of necrotic cell death, and pyronecrosis, a variant of the highly inflammatory cell death process pyroptosis (108, 109). PVL, among many other toxins, have been shown to exacerbate lung damage through the induction of necroptosis (109) and PVL positive strains have a strong association with necrotising pneumonia (110). Although inducing necroptosis appears to be counterintuitive to the mechanism of intracellular survival, this may represent an alternative mechanism of immune evasion which could benefit extracellular *S. aureus* by overwhelming and eradicating local immune cells.

## Potential Host Factors Involved in *S. aureus* Autophagy-Mediated Intracellular Survival

No specific host factor has yet been singled out as integral to autophagy-mediated *S. aureus* intracellular survival in PMN. However, as this autophagy dependent mechanism has been shown to delay apoptosis in PMN, any host factors that possess dual functions in both autophagy and apoptosis should be considered as potential candidates co-opted by *S. aureus* to facilitate intracellular survival. The antiapoptotic protein Mcl-1 has the potential to be a host-specific factor involved in autophagy-mediated intracellular survival of *S. aureus*. As discussed, *S. aureus* intracellular survival in PMN leads to the upregulation of Mcl-1 and this correlates with an anti-apoptotic phenotype in PMN (21). This anti-apoptotic phenotype occurs alongside a block in autophagic flux, creating an intracellular niche for *S. aureus*. Consequently, disrupted host autophagy and elevated Mcl-1 levels could result in the delayed apoptotic phenotype thus creating a suitable intracellular niche for *S. aureus* to reside.

The transcription factor p53 plays a regulatory role in apoptosis and is activated as part of the host response to cellular stress. Additionally, p53 can be both pro-autophagic and anti-autophagic depending on its cellular location (111, 112). Under homeostatic conditions, p53 is retained in the nucleus where it can exert pro-autophagic effects. Translocation from the nucleus to the cytoplasm leads to pro-apoptotic effects and usually occurs during cellular stress (113, 114). During *S. aureus* intracellular survival in primary human PMN, p53 was primarily located in the nucleus, suggesting it played a pro-autophagic role in this model (21). Damage-Regulated Autophagy Monitor (DRAM) is a direct translational product of p53 and has been shown to play a role in both autophagy activation and apoptosis (115, 116). Gene and protein expression of p53 and DRAM were increased during autophagy-mediated *S. aureus* intracellular survival in PMN (21). Furthermore, transcription of both were reduced after autophagy inhibition, suggesting that the autophagy pathway and the p53/DRAM pathway are directly linked during *S. aureus* intracellular survival in PMN. Additionally, intracellular survival of *S. aureus* was reduced after inhibition of p53 using the p53 inhibitor pifithrin-α. This was accompanied by a subsequent reduction in DRAM gene transcription. These results suggest that an active p53/DRAM pathway is necessary for efficient autophagy-mediated *S. aureus* intracellular survival. Under HIV infection, CD4+ T cells show enhanced p53 dependent gene expression of DRAM which leads to the induction of host autophagy (117). However, in this model, normal autophagic flux was detected, and increased DRAM expression was associated with enhanced lysosomal membrane permeabilization and cell death. In the intracellular survival of *S. aureus*, PMN could potentially be protected from this p53 dependent DRAM induced cell lysis by Mcl-1. However, PMN could still show DRAM induced autophagy which the bacterium could manipulate for its own survival. Over expression of DRAM in a zebrafish model led to hyperactivation of host autophagy leading to enhanced mtb clearance through the enhancement of host xenophagy (118). Whilst mtb is susceptible to xenophagy, *S. aureus* manipulates this pathway and so enhanced autophagy would be beneficial to the pathogen, potentially highlighting the importance of DRAM in positively inducing autophagy mediated intracellular survival of *S. aureus* in PMN.

Although the p53/DRAM pathway has been highlighted as a potential target of *S. aureus* to enhance host autophagy, it is likely that the highly adept *S. aureus* can manipulate other pathways to achieve the same outcome. Extracellular conserved chromatin-binding nuclear protein high mobility group box 1 (HMGB1) acts as a DAMP in infection and has been shown to enhance pathology in an *S. aureus* pneumonia model associated with a massive influx of PMN (119). However, HMGB1 was shown not to affect the influx of PMN, nor did it affect bacterial clearance yet when blocked, with anti-HMGB1, did lead to significant reductions in IL-1β production. Conversely, PMSα, a staphylococcal toxin associated with intracellular survival, has been shown to attenuate HMGB1 binding to TLR4 leading to suppression of the pro-inflammatory cytokines TNF and IL-6 in THP-1 macrophages (120). Together these results implicate the involvement and manipulation of HMGB1 in *S. aureus* infection which has yet to be investigated in the context of intracellular survival. Interestingly, intracellular HMGB1 can also facilitate activation of the autophagy pathway and thus should be considered in autophagy mediated intracellular survival of *S. aureus*. Intracellular HMGB1 contributes to the protection of mice from endotoxemia and bacterial infection by positively mediating the xenophagy pathway in peritoneal macrophages and was found to be a crucial regulator of autophagosome formation during bacterial challenge *in vivo* (121). LC3 processing was reduced in HMGB1-low peritoneal macrophages from mice after *L. monocytogenes* infection, indicating that HMGB1 is crucial for activation of bacteria-induced autophagy. Loss of HMGB1 severely reduces bacterial clearance in this model since autophagy is needed to kill *L. monocytogenes*. It is tempting to speculate that intracellular HMGB1 may also induce autophagy after *S. aureus* exposure in PMN. HMGB1 is released in bronchiolar lavage fluid and contributes to lung injury in a murine model of *S. aureus* pneumonia (119). This may be due to the enhanced autophagic response elicited by platelet derived HMGB1 which has been shown to enhance autophagosome formation in PMN from patients with acute myocardial infractions (122). This study also showed that HMGB1 was able to prolong PMN survival whilst preventing mitochondrial potential depletion which, in combination with the induction of autophagy, is a strikingly similar phenotype to that seen in *S. aureus* infection of PMN.

## Bacterial Factors Involved in *S. aureus* Autophagy-Mediated Intracellular Survival

*S. aureus* clearly changes the intracellular landscape of the host cell to benefit its own survival by co-opting autophagosomes and host factor gene expression to reshape the autophagic and apoptotic fate of PMN for its own benefit. However, bacterial factors produced by *S. aureus* to manipulate PMN in this context represent a major gap in our current understanding of the intracellular life cycle of this bacterium. Even within the broader context of autophagy mediated *S. aureus* intracellular survival in professional and non-professional phagocytes, little is known. It is likely, given the pleiotropic nature and redundancy of several staphylococcal toxins, such as the PSMs, that multiple factors are involved but to date, only a few factors have been identified within a handful of cell types. For instance, several reports have highlighted the involvement of the *agr* regulated toxin, hla, in the induction of host autophagy. Treatment with hla alone was capable of inducing LC3 decorated vacuoles within CHO cells (67). Vacuoles containing hla mutants failed to recruit LC3 to these vacuoles in contrast to the hla expressing strain. In a follow up study, the same authors found that hla could induce LC3 decorated filaments in a Rab7 and Rab1b dependent manner which is in inhibited by cAMP (68). Studies in HACAT cells have also suggested that hla is capable of inducing autophagy possibly due to a drop in ATP/AMP ratio in response to membrane perforation (123). It is reasonable to suggest that hla expression may also induce autophagy to the same effect in neutrophils which could provide *S. aureus* a protective niche in which to thrive. Besides its effect on the autophagy pathway, hla has been shown to inhibit macrophage efferocytosis potentially to protect its intracellular niche within PMN from phagocytosis and killing by host macrophages (124). Regardless of the effect hla has on inducing host autophagy, it is unclear what causes the failure of host autophagosomes to mature into autophagolysosomes. However, one study showed that *S. aureus* was capable of disrupting autophagic flux through the expression of Immunodominant surface antigen B (IsaB) within Hela cells, THP-1 macrophages and *in vivo* using a skin infection model (125). Deletion of IsaB led to increased autophagic flux which was effectively reduced by recombinant IsaB thus implicating its role in the manipulation of host autophagy for intracellular survival. The role IsaB plays in subverting PMN has not yet been investigated but could potentially act as a crucial factor in achieving the abhorrent autophagic and apoptotic phenotype seen within PMN under *S. aureus* infection. Further research is needed to determine the bacterial factors involved in manipulating host autophagy by intracellular *S. aureus* across all cell types but particularly within PMN.

## Can We Treat *S. aureus* Infection by Manipulating Autophagy?

The manipulation of host autophagy by *S. aureus* for intracellular survival opens the possibility for targeting the autophagy pathway as an additive or alternative treatment for *S. aureus* infection, particularly in cases where antibiotic treatment is not successful. Recently, high throughput studies have begun to assess potential host directed therapies and host targets that can be used against intracellular *S. aureus* (126, 127). One such study used a shRNA screen in HeLa cells to assess host factors involved in *S. aureus* infection which highlighted several potential host targets that could lead to increased host cell viability including the pro-autophagic gene ATG10, though this target was not further assessed. However, as this review has highlighted, intracellular survival of *S. aureus* likely varies among strains, target host cell type and is likely dependent on bacterial/host gene expression. The complexity of these factors, and their relationship to one another, will therefore present a difficult challenge in finding a reliable target for host directed therapy. Nevertheless, drugs targeting host cells and pathways can lead to improved disease outcome in *S. aureus* infection, several of which that target the autophagy pathway.

The anti-malarial drug chloroquine, which can inhibit autolysosome formation, has been shown to enhance the killing of intracellular *S. aureus* using the antibiotic, levofloxacin, when co-administered in THP-1 macrophages (128). Although the effectiveness of chloroquine has not been attributed to its anti-autophagic ability, its clinically approved derivative, hydroxychloroquine, has been shown to modulate autophagy within a subset of patients as part of ongoing preclinical studies for anti-tumour treatments (129). Therefore, it can be speculated that this enhanced killing can be attributed to the effect’s chloroquine exerts on host autophagy and thus should be investigated in the context of autophagy mediated intracellular survival of *S. aureus* within PMN. Another autophagy inhibitor, Dorsomorphin, has been used in several *in vitro* and *in vivo* studies to inhibit AMPK, a key inducer of host autophagy (130). Recently, Dorsomorphin has been shown *in vitro* to reduce intracellular burden of *S. aureus* and increase cell viability in both HeLa and HUVEC cells which could potentially yield similar results in PMN through the obstruction of host autophagy (65). However, it should be noted that dorsomorphin can have off target effects which may likely hinder its potential use in a clinical setting (130). Lastly, the autophagy inhibitor, 3-MA, has recently been shown to protect against *S. aureus* infection in a BALB/c lung infection model (131). In this study, Raw264.7 macrophages showed increased bacterial killing of *S. aureus* when treated with 3MA which was associated with the reduction of the autophagic markers LC3-II and Beclin-1. *In vivo*, 3MA treated mice showed comparable effects to vancomycin treatment which showed reduced lung CFU and increased numbers of CD11b^+^F4/80^+^ macrophages versus control mice. The authors reasoned that *S. aureus* manipulates autophagy within host macrophages for its own gain and so when treated with the autophagy inhibitor, 3MA, efficient killing of the bacterium by host macrophages was restored. However, given the widespread inhibition 3MA would have across all cell types, it could be theorized that autophagy inhibition also restored neutrophil killing leading to overall improvement in infection outcome. In all, targeting host autophagy has yielded some encouraging results in the treatment of *S. aureus* infection in combination with antibiotics as well as a standalone treatment.

It is possible that existing clinically approved autophagy inhibitors could be re-purposed to treat *S. aureus* chronic infection. However, inhibiting or blocking host autophagy could have unwanted side effects and so, would need to be approached with caution. Maurer et al., generated a breed of mice with a partial knockdown of the critical LC3 processing protein ATG16L1 leading to a reduced autophagic phenotype across all tissues and cell types examined (132). ATG16L1 hypomorphic (ATG16L1^HM^) mice infected with *S. aureus* were shown to possess a significantly enhanced susceptibility to hla leading to increased mortality (133). This increased susceptibility of ATG16L1^HM^ mice was not a result of the altered autophagy phenotype in macrophages or dendritic cells but rather due to the autophagy reduced endothelial cells. This was accredited to an inability of the ATG16L1^HM^ mice endothelial cells to mediate hla tolerance through the autophagy pathway which under WT conditions would limit hla toxicity. Interestingly, ATG16L1^HM^ mice were shown to have significantly enhanced survival against hla mutants compared to WT mice indicating the fine balance autophagy plays in *S. aureus* infection. As such, this study demonstrates the risk of inhibiting host autophagy and highlights the need for greater understanding of the relationship of *S. aureus* and the autophagy pathway as well as any bacterial factors involved. Of course, blanket autophagy inhibition may also inadvertently block the clearance of another pathogen. For example, treatment of cystic fibrosis patients with the antibiotic azithromycin led to opportunistic mycobacterial infections; azithromycin blocked autolysosome formation and therefore the degradation of mycobacteria within autophagosomes. It is therefore critical that these considerations are taken into account when considering clinical inhibition of autophagy. However, despite these potential downfalls, inhibition of host autophagy remains an alluring option to combat *S. aureus* infection.

## Conclusion

*S. aureus* is a formidable foe which has developed a vast array of immune evasion mechanisms that enable its survival and persistence. Of these mechanisms, *S. aureus* intracellular survival represents one of the most advantageous tactics to subvert the host immune system. Such a mechanism offers both stealth and protection from host bacterial sensors, killing and even from antibiotics due to poor membrane permeability and the formation of intracellular SCVs (5, 134, 135). Intracellular survival is clearly of great benefit to *S. aureus* as it has been shown in numerous cell types with various mechanisms employed in both a strain and cell dependent manner. Of these mechanisms, the manipulation of host autophagy appears to play a central role in both short- and long-term intracellular survival with many professional and non-professional phagocytes shown to facilitate intracellular *S. aureus* in an autophagy dependent manner. Among these cell types, PMN represent a highly advantageous target of *S. aureus* creating an unlikely intracellular niche to shield the bacterium from extracellular threats whilst providing safe passage to secondary sites for further infection. Despite the hazardous intracellular conditions found within PMN, *S. aureus* can survive intracellularly and can masterfully manipulate the host autophagy pathway, a process designed to remove intracellular pathogens. In doing so, *S. aureus* can subvert the constitutively active apoptosis pathway in PMN to prevent destruction of its intracellular niche whilst also protecting itself from the degradative process of apoptosis and subsequent macrophage efferocytosis.

Whilst several host and bacterial factors have been identified in this context, more research is required with a focus needed in the context of host directed therapies that represent a massively important field of research currently underrepresented. Future studies should aim to give greater insight into the molecular pathways engaged within PMN by *S. aureus* as well as any bacterial factors involved whilst also focusing on increased *in vivo* studies and clinical observations of novel host directed therapies. Nevertheless, intracellular survival and host autophagy is a crucial factor in *S. aureus* infection which has the potential to yield powerful next generation therapeutics capable of disarming the pathogen of one of its most important immune evasion tactics.

## Author Contributions

MM and RM conceived the idea of the manuscript. EV and MM wrote the manuscript. RM edited and added invaluable insights to the manuscript. All authors contributed to the article and approved the submitted version.

## Funding

Associated work in the lab was supported by a Science Foundation Ireland Investigator Award (15/IA/3041) and a Wellcome Investigator Award (202846/Z/16/Z) to RM.

## Conflict of Interest

The authors declare that the research was conducted in the absence of any commercial or financial relationships that could be construed as a potential conflict of interest.
